# Dynamic Transitions of Epilepsy Waveforms Induced by Astrocyte Dysfunction and Electrical Stimulation

**DOI:** 10.1155/2020/8867509

**Published:** 2020-11-16

**Authors:** Honghui Zhang, Zhuan Shen, Qiangui Zhao, Luyao Yan, Lin Du, Zichen Deng

**Affiliations:** ^1^School of Mathematics and Statistics, Northwestern Polytechnical University, Xi'an 710072, China; ^2^School of Aeronautics, Northwestern Polytechnical University, Xi'an 710072, China

## Abstract

Experimental studies have shown that astrocytes participate in epilepsy through inducing the release of glutamate. Meanwhile, considering the disinhibition circuit among inhibitory neuronal populations with different time scales and the feedforward inhibition connection from thalamic relay nucleus to cortical inhibitory neuronal population, here, we propose a modified thalamocortical field model to systematically investigate the mechanism of epilepsy. Firstly, our results show that rich firing activities can be induced by astrocyte dysfunction, including high or low saturated state, high- or low-frequency clonic, spike-wave discharge (SWD), and tonic. More importantly, with the enhancement of feedforward inhibition connection, SWD and tonic oscillations will disappear. In other words, all these pathological waveforms can be suppressed or eliminated. Then, we explore the control effects after different external stimulations applying to thalamic neuronal population. We find that single-pulse stimulation can not only suppress but also induce pathological firing patterns, such as SWD, tonic, and clonic oscillations. And we further verify that deep brain stimulation can control absence epilepsy by regulating the amplitude and pulse width of stimulation. In addition, based on our modified model, 3 : 2 coordinated reset stimulation strategies with different intensities are compared and a more effective and safer stimulation mode is proposed. Our conclusions are expected to give more theoretical insights into the treatment of epilepsy.

## 1. Introduction

Epilepsy has been the second worldwide neurological disorder that affects over 65 million people [1]. As a type of generalized epilepsy, absence epilepsy is featured by periodic 2-4 Hz SWDs in electroencephalography (EEG) recordings, mainly occurring in children and accompanying a brief impairment of consciousness in seizures [2–4]. Epileptic tonic and clonic seizures are also the primary generalized seizures, seriously affecting the patients' life [5–7]. The EEG of patients with tonic seizures shows high-frequency and low-amplitude rapid discharge activities, while clonic seizures exhibit low-frequency and high-amplitude slow-wave oscillations [8, 9]. Furthermore, the evolution of tonic to clonic seizures has been observed clinically [8, 9]. Since the causes of epilepsy are complex and the pathogenesis is still not entirely clear, it has become a major topic for many neuroscientists.

It is traditionally believed that epilepsy mainly results from the imbalance of excitatory and inhibitory factors in the nervous system, such as excitatory or inhibitory synaptic dysfunction and neurotransmitter imbalance [10–12]. Some scholars have pointed out that absence epilepsy was initiated in the cortex [13], some believed that it originated from thalamus [14, 15], while others have attributed it to the abnormality of cortical thalamic network [16, 17]. Moreover, many important advances have shown that astrocytes are involved in neuronal firing behaviors [18–21] as well as epilepsy [22–26]. Steinhäuser and Seifert pointed out that the abnormal expression of potassium channels, glutamate receptors, and gap junctions in astrocytes can induce epileptic discharges [22, 23]. Crunelli et al. proposed that astrocytes can provide an important basis for epilepsy treatment [26]. Tian et al. showed that glutamate released by astrocytes stimulates neuronal excitatory receptors, possibly causing hyperexcitatory epileptic discharges [27–30]. Li et al. had given insight into the effect of astrocyte Ca^2+^ oscillation on the generation, spread, and inhibition of epilepsy from the perspective of dynamics [31–33].

Researches based on computational models, including mesoscopic neuron population models [34], mean field models [35, 36], and neural field models [37, 38], have greatly promoted people's understanding of epilepsy from different levels. As one of the representatives of the neural field model, Taylor model can perfectly simulate the 2-4 Hz SWD that characterizes absence seizures [37, 38]. Recently, scholars have proposed many improved models to study absence epilepsy [39, 40]. For examples, Fan et al. investigated the combined effects of feedforward inhibition and excitation in thalamocortical circuit on the transition behaviors of epileptic seizures by introducing the feedforward excitation from thalamic relay nucleus to excitatory pyramidal neuronal into the thalamocortical network [39]. Liu et al. constructed another modified thalamocortical network by considering a disinhibition neural population [40].

It was estimated that one-third of epilepsy patients have difficulties getting good treatment with antiepileptic drugs [41]. Surgical resection may be a good treatment choice just for a minority of patients [42]. Finding new treatments is still an urgent problem in the field of antiepileptic research. Taylor et al. found that the transitions between normal and epileptic states can be induced by single-pulse stimulation and further studied the effect of stimulation on SWD [38]. Ge et al. presented a robust closed-loop control of SWD based on a thalamocortical computational model of absence epilepsy [43]. Wang et al. tried different stimulation strategies, including deep brain stimulation (DBS), 1 : 0 coordinated reset stimulation (CRS), and 3 : 2 CRS [44]. These methods are all attempts based on theoretical models, but they can give some scientific enlightenments to the direction of clinical treatment.

In this paper, we provide a possibility that astrocytes are involved in epileptic seizure based on a modified neural field model, which will be introduced in detail in the next section. Then, we are dedicated to studying the transition dynamics induced by astrocyte dysfunction and focus on the control effects after different stimulations applying to thalamic neuronal population. Finally, we give the discussions and conclusions.

## 2. Materials and Methods

### 2.1. Description of the Modified Neural Field Model

Pi et al. proved that there are mutual effects among different inhibitory neurons with different time scales [45]. Beverlin et al. showed that feedforward inhibition from thalamic relay nucleus (TC) to inhibitory interneuron has even more stronger functions on the cortex than the feedforward excitation from TC to excitatory pyramidal neuronal (PY) [46]. To the best of our knowledge, although Fan et al. and Liu et al. had considered the above-mentioned physiological phenomena in their computational models separately [39, 40], no scholars have considered these two facts at the same time. Motivated by these, we propose a more comprehensive model to systematically investigate the effects of important model parameters on the transition behaviors of epileptic seizures including tonic-clonic and absence seizures.

**Figure 1:**
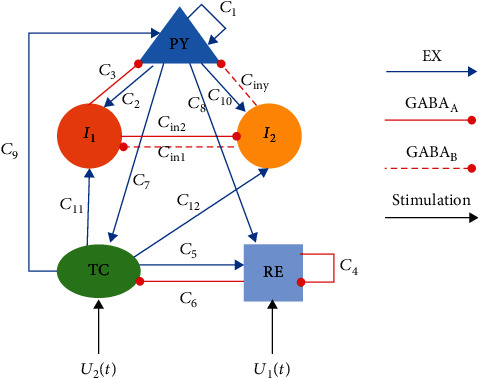
The topological structure of the model, which is composed of excitatory pyramidal neuronal (PY), inhibitory interneuron with different time scales (*I*_1_, *I*_2_), thalamic relay nucleus (TC), and thalamic reticular nucleus (RE). The blue lines indicate the excitatory projection of glutamate receptors, while red solid lines and red dashed lines represent the inhibitory projection mediated by GABA_A_ and GABA_B_, respectively. *U*_1_(*t*) and *U*_2_(*t*) are the stimulus on RE and TC.

The improved model is composed of cortical subnetwork and thalamus subnetwork. The former is composed of excitatory pyramidal neuronal (PY) and inhibitory interneuron with different time scales (*I*_1_, *I*_2_), while the latter is formed from the thalamic relay nucleus (TC) and thalamic reticular nucleus (RE). The excitatory projections of glutamate receptors are represented by blue lines, and the inhibitory projections mediated by GABA_A_ and GABA_B_ are denoted by red solid lines and red dashed lines, respectively. In addition, the stimulus considered are applied to RE and TC, which are indicated by *U*_1_(*t*) and *U*_2_(*t*), respectively (see Figure 1). Therefore, the nonlinear system can be described as below:

(1) $$\begin{array}{c} \frac{d\text{PY}}{dt}=\sigma_{1}\left(h_{\text{PY}}-\text{PY}+C_{1}f\left(\text{PY}\right)-C_{3}f\left(I_{1}\right)+C_{9}f\left(\text{TC}\right)-C_{\text{iny}}f\left(I_{2}\right)\right),\\ \frac{dI_{1}}{dt}=\sigma_{2}\left(h_{I_{1}}-I_{1}+C_{2}f\left(\text{PY}\right)-C_{\text{in}1}f\left(I_{2}\right)+C_{11}f\left(\text{TC}\right)\right),\\ \frac{dI_{2}}{dt}=\sigma_{3}\left(h_{I_{2}}-I_{2}+C_{10}f\left(\text{PY}\right)-C_{\text{in}2}f\left(I_{1}\right)+C_{12}f\left(\text{TC}\right)\right),\\ \frac{d\text{TC}}{dt}=\sigma_{4}\left(h_{\text{TC}}-\text{TC}-C_{6}g\left(\text{RE}\right)+C_{7}f\left(\text{PY}\right)\right)+U_{2}\left(t\right),\\ \frac{d\text{RE}}{dt}=\sigma_{5}\left(h_{\text{RE}}-\text{RE}-C_{4}g\left(\text{RE}\right)+C_{5}g\left(\text{TC}\right)+C_{8}f\left(\text{PY}\right)\right)+U_{1}\left(t\right), \end{array}$$

where PY, *I*_1_, *I*_2_, TC, and RE denote different types of populations and *σ*_1_, *σ*_2_, *σ*_3_, *σ*_4_, and *σ*_5_ are the time scales, while *h*_PY_, *h*_*I*_1__, *h*_*I*_2__, *h*_TC_, and *h*_RE_ are external inputs. *C*_*i*_(*i* = 1, 2, 3, ⋯, 9) are strength of coupling between different neuron populations. *U*_1_(*t*) and *U*_2_(*t*) are the stimulus on RE and TC. *f*(∙) and *g*(∙) are transfer functions [37, 38], which can be expressed as follows:

(2) $$\begin{array}{c} f\left(x\right)=\frac{1}{1+\varepsilon^{-x}},\\ g\left(y\right)=ay+b, \end{array}$$

where *x* = PY, *I*_1_, *I*_2_, and *y* = TC, RE. *ε* and *a* determine the steepness of the two transfer functions, respectively, and *b* is a constant. In addition, unless noted otherwise, values of model parameters in our simulations are consistent with previous studies [37–40]. New parameters are set within a reasonable range to study the transition behaviors of epileptic seizures (see Table 1).

### 2.2. Numerical Simulation Methods and Data Analysis

In this paper, the differential equations are solved by the standard fourth-order Runge-Kutta integration method under the MATLAB (MathWorks, USA) simulating environment. And the time window is 30 s with integration step 1 ms. The time series of each cortical neuron population are averaged and used for data analysis (i.e., (PY + *I*_1_ + *I*_2_)/3). The bifurcation diagrams are plotted by calculating the stable local minimum and maximum values of (PY + *I*_1_ + *I*_2_)/3 as parameters gradually vary. At the same time, we provide dynamical analysis corresponding to some typical state transitions by using the continuation package, AUTO in XPPAUT (available from http://www.math.pitt.edu/bard/xpp/xpp.html). In addition, we acquire normalized power spectral density by utilizing the fast Fourier transform and the maximum peak frequency is defined as the dominant frequency of the oscillation.

## 3. Results

### 3.1. Transition Dynamics Induced by Astrocyte Dysfunction

In the central nervous system, there are many cell types capable of expressing glutamate carriers, such as astrocytes and neurons [47]. Only when glutamate overflows, the neuron carrier can play a role. That is to say, the uptake of glutamate by astrocytes is the main way to clear glutamate in the intercellular space [47, 48]. The weaker the uptake capacity of astrocytes, the greater the uptake capacity that neurons need to bear, and the stronger the coupling connection. In addition, electrophysiological experiments have shown that epilepsy is caused by abnormal information exchange between the cortex and thalamus circuits [49, 50]. In our model, it is considered that the cortex subnetwork and the thalamus subnetwork are mainly mediated by glutamate receptors [51]. Inspired by these, in this section, we provide a possibility that astrocytes are involved in epileptic seizure and we are dedicated to studying the transition dynamics induced by astrocyte dysfunction. More precisely, we mainly investigate the effect of parameter *C*_7_.

**Figure 2:**
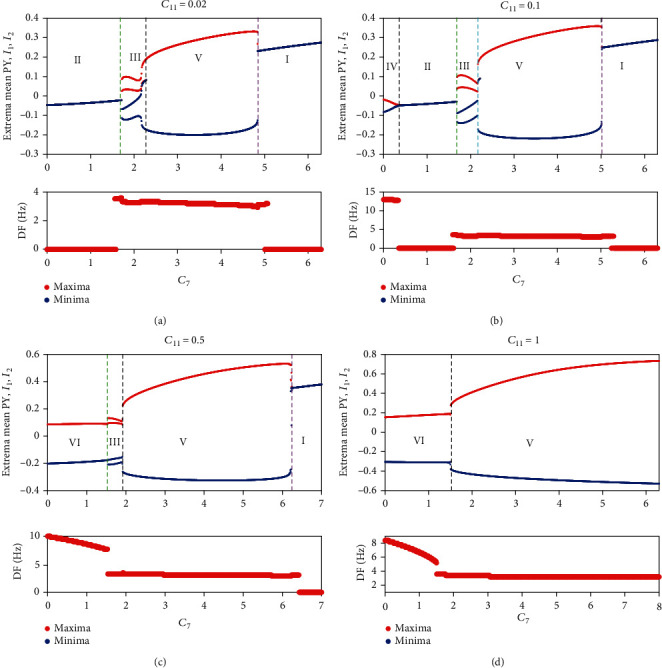
The bifurcation diagram (upper panels) and their corresponding dominant frequencies (lower panels) induced by *C*_7_, when *C*_11_ is set to 0.02 (a), 0.1 (b), 0.5 (c), and 1 (d), respectively. Six firing modes can be observed such as I (high saturated state), II (low saturated state), III (SWD), IV (tonic), V (low-frequency clonic), and VI (high-frequency clonic). The dominant frequencies of states I and II are 0 Hz, of states III and V are between 2 Hz and 4 Hz, and of state VI are between 5 Hz and 10 Hz, and the dominant frequencies of state IV are up to 13 Hz.

**Figure 3:**
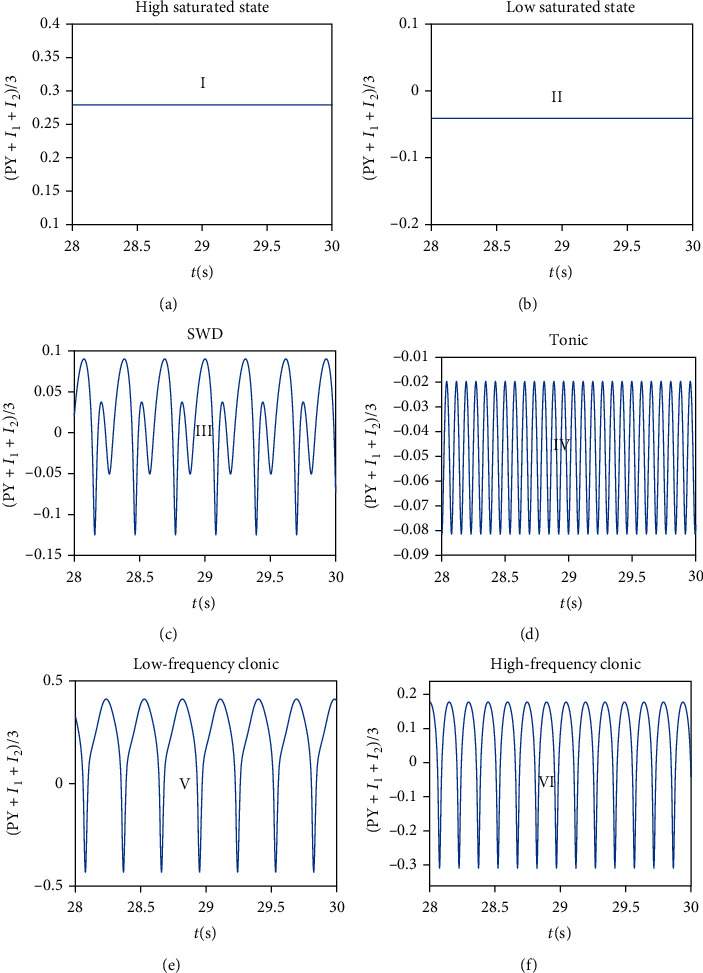
The time series of different firing modes in Figures 2 and 4: (a) high saturated state, (*C*_7_, *C*_11_) = (6, 0.1); (b) low saturated state, (*C*_7_, *C*_11_) = (1, 0.1); (c) SWD, (*C*_7_, *C*_11_) = (2, 0.1); (d) tonic, (*C*_7_, *C*_11_) = (0.02, 0.1); (e) low-frequency clonic, (*C*_7_, *C*_11_) = (2, 1); (f) high-frequency clonic, (*C*_7_, *C*_11_) = (1, 1).

Firstly, we set *C*_11_ = 0.1, and the remaining parameters are consistent with Table 1. Rich firing modes can be captured as the bifurcation parameter *C*_7_ varies (see Figure 2(b)). The more functional the astrocytes are, the less glutamate the neurons absorb; that is, the smaller *C*_7_ is, then the brain will exhibit tonic oscillation (i.e., IV). If astrocytes function normally, the system will show normal low saturated state (i.e., II). However, when the function of astrocytes is inhibited or damaged, the glutamate absorbed by neurons exceed the normal value, and the system shows SWD oscillation (i.e., III). With the further increase in absorption, the system transitions from SWD to low-frequency clonic oscillation (i.e., V). If glutamate accumulates in a large amount, neurons will be overexcited, and the system will transit to high saturation state (i.e., I). The oscillation dominant frequencies of all discharge modes can be observed in the figure's lower panel (see Figure 2(b)) and the time series of different firing modes are shown in Figure 3.

Secondly, without loss of generality, we display the dynamical analysis corresponding to the state transitions in Figure 2(b) (see Figure 5). For smaller *C*_7_ (i.e., *C*_7_ < 0.24), there is a monostable region consisting of one unstable fixed point and one stable limit cycle; hence, all the simulations converge to the stable limit cycle, and system exhibits tonic oscillations. Then, the tonic oscillation disappears and the low saturated state appears following a supercritical Hopf bifurcation (i.e., HB_1_). Next, with the further increase of *C*_7_, the system successively undergoes the fold of cycle bifurcation (LPC_1_) and the subcritical Hopf bifurcation (HB_2_) and evolves into a bistable region (i.e., 1.69 ≤ *C*_7_ ≤ 2.16). When 2.16 ≤ *C*_7_ ≤ 3.47, the system is in monostable state until the subcritical Hopf bifurcation (HB_3_) appears. Immediately afterwards, the system transits to the second bistable area (i.e., 3.47 < *C*_7_ ≤ 5.01). Last, all the simulations converge to the steady high saturated state due to the fourth fold limit cycle bifurcation (LPC_4_). In addition, it is worth noting that during our simulations, the initial values are set to [0, 0, 0, 0, 0]. In the above two bistable regions, the initial values are close to the side of separatrix near stable limit cycle, so the system will converge to the periodic oscillation (see Figure 2(b)).

Thirdly, considering the complexity of neural networks, we give the dynamic transition phenomenon induced by *C*_7_ when *C*_11_ is set to 0.02 (see Figure 2(a)), 0.5 (see Figure 2(c)), and 1 (see Figure 2(d)), respectively. If *C*_11_ = 0.02, tonic oscillation will disappear. For the larger *C*_11_, the system can exhibit high-frequency clonic (i.e., VI), whose dominant frequency is between 5 Hz and 10 Hz (see Figure 2(c)). However, only high-frequency clonic and low-frequency clonic can be observed in the inspection window when *C*_11_ = 1.

**Figure 4:**
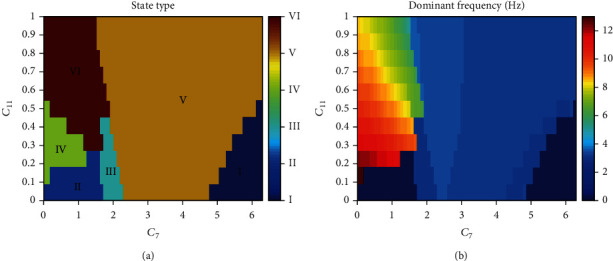
Different firing states (a) and variations of corresponding dominant frequencies (b) are shown on the parameter plane (*C*_7_, *C*_11_), where we can observe rich firing states such as I (high saturated state), II (low saturated state), III (SWD), IV (tonic), V (low-frequency clonic), and VI (high-frequency clonic). In addition, the dominant frequencies of states I and II are 0 Hz, of states III and V are between 2 Hz and 4 Hz, and of state VI are between 5 Hz and 10 Hz, while the dominant frequencies of state IV are greater than 10 Hz.

**Figure 5:**
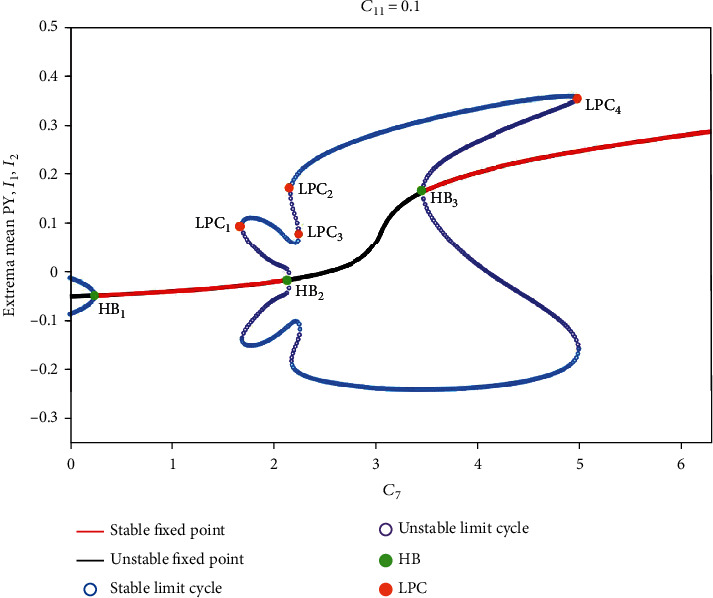
Dynamical analysis corresponding to the state transitions in Figure 2(b). As *C*_7_ increases, the system successively undergoes the supercritical Hopf bifurcation (HB_1_), the fold of cycle bifurcation (LPC_1_), the subcritical Hopf bifurcation (HB_2_), LPC_2_, LPC_3_, the subcritical Hopf bifurcation (HB_3_), and LPC_4_.

Lastly, in order to observe the interaction between *C*_7_ and *C*_11_ generally, we show the state type and dominant frequency when *C*_7_ and *C*_11_ both change (see Figure 4). Rich firing states can be seen by us, such as I (high saturated state), II (low saturated state), III (SWD), IV (tonic), V (low-frequency clonic), and VI (high-frequency clonic). With the enhancement of feedforward inhibition connection, SWD, tonic, and saturated state will disappear. In addition, the dominant frequencies of states I and II are 0 Hz, states III and V are between 2 Hz and 4 Hz, and state VI are between 5 Hz and 10 Hz, while the dominant frequencies of state IV are greater than 10 Hz. Perhaps, epilepsy can be controlled by regulating the uptake of glutamate by astrocytes.

### 3.2. Control of Single-Pulse Stimulation

As a gate controller of the thalamus and an important part of the cortex-thalamic circuit, RE plays an extremely important role in the activation of absence seizures [52, 53]. It has been reported that RE is involved in tonic seizures and clonic seizures [54, 55], and different types of seizures correspond to different levels of RE's excitability. In addition, single-pulse stimulation has been suggested to prematurely terminate the seizure [18, 56]. Hence, in the following sections, we focus on the dynamic transitions after single-pulse stimulation acting on RE.

**Figure 6:**
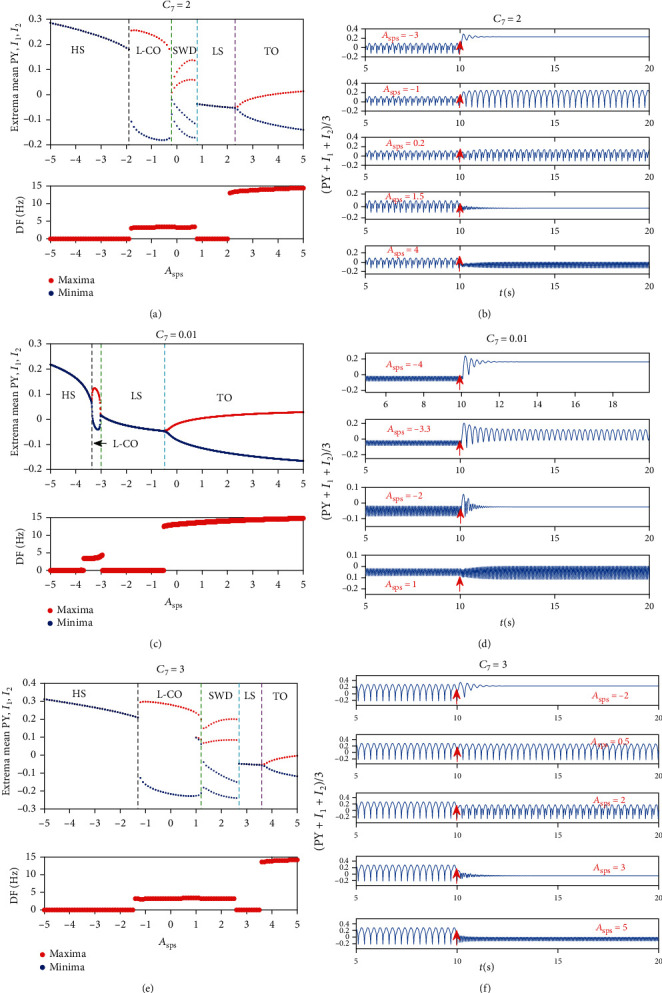
The bifurcation diagram (upper panel of (a), (c), and (e)), their corresponding dominant frequencies (lower panel of (a), (c), (e)), and the specific time series (b, d, f) after single-pulse stimulation acting on RE at *t* = 10 s when the system is in SWD (a, b), tonic (c, d), and low frequency clonic (e, f) originally. For clarity, TO (tonic), SWD, LS (low saturated state), HS (high saturated state), l-CO (low-frequency clonic oscillations), and the strength of stimulation (*A*_sps_) are indicated by text labels in each figure.

Firstly, we care the effects of single-pulse stimulation when the system is in pathological states. Obviously, rich dynamic transitions can be captured after single-pulse stimulation acting on RE at *t* = 10 s when the system is in SWD originally (see Figure 6(a)). It is clear that low-frequency clonic and high saturated state can be induced by applying a negative stimulus inputs to RE. On the contrary, when stimulus inputs are positive, SWD, low saturated state, and tonic appear successively as the strength of stimulation increases. The corresponding dominant frequencies and the specific time series also are plotted in Figures 6(a) and 6(b). If we make the initial states to be tonic, we can easily see that tonic oscillation can transit to high saturated state, low-frequency clonic, low saturated state, and even tonic (see Figures 6(c) and 6(d)). Furthermore, when the initial state is low-frequency clonic, the phenomenon is more fascinating. Similar to the case where the initial state is SWD, five firing modes can be induced (see Figures 6(e) and 6(f)). The specific time series diagram for each transition is shown as an example in Figures 6(b), 6(d), and 6(f), and the corresponding strength of stimulation is set to -2, 0.5, 2, 3, 5, respectively (see Figure 6(f)).

**Figure 7:**
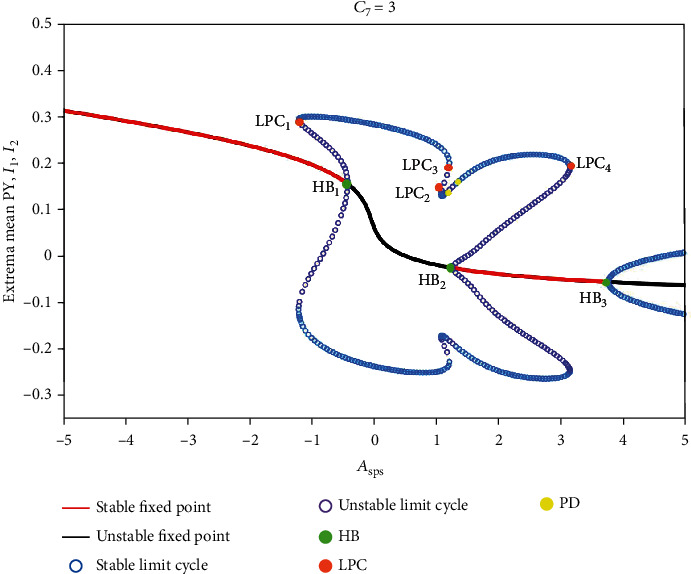
Dynamical analysis corresponding to the state transitions in Figure 7(e). As *A*_sps_ increases, the system successively undergoes the fold of cycle bifurcation (LPC_1_), the subcritical Hopf bifurcation (HB_1_), LPC_2_, LPC_3_, period doubling bifurcation (PD_1_), the subcritical Hopf bifurcation (HB_2_), PD_2_, LPC_4_, and the supercritical Hopf bifurcation (HB_3_).

For more details, we also display the dynamical analysis corresponding to the state transitions in Figure 6(e) (see Figure 7). For the much small *A*_sps_ (i.e., *A*_sps_ < −1.22), there is a monostable region consisting of the stable fixed points, the system converges to stable high saturated state. For the less small *A*_sps_ (i.e., −1.22 ≤ *A*_sps_ < −0.44), the system transits to the first bistable region between the fold of cycle bifurcation (LPC_1_) and the subcritical Hopf bifurcation (HB_1_). Then, the system successively undergoes LPC_2_, LPC_3_, period doubling bifurcation (PD_1_), and the subcritical Hopf bifurcation (HB_2_) as *A*_sps_ increases. At the same time, the system is always in a monostable region (i.e., −0.44 ≤ *A*_sps_ < 1.20). Next, the second bistable area appears, which is between the subcritical Hopf bifurcation (HB_2_) and LPC_4_. And the system transits to a monostable region following LPC_4_. Last, all the simulations converge to the tonic oscillations due to the supercritical Hopf bifurcation (HB_3_). Similarly, the initial values are close to the side of separatrix near stable limit cycle in the above two bistable regions, and the system exhibits the periodic oscillations (see Figure 6(e)).

**Figure 8:**
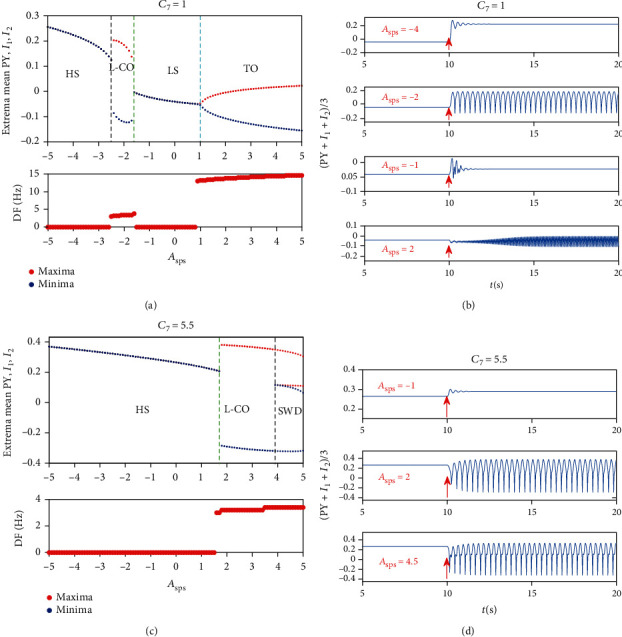
The bifurcation diagram (upper panel of (a, c)), their corresponding dominant frequencies (lower panel of (a, c)), and the specific time series (b, d) after single-pulse stimulation acting on RE at *t* = 10 s when the system is in low saturated state (a, b) and high saturated state (c, d) originally. Similarly, HS (high saturated state), l-CO (low-frequency clonic oscillations), LS (low saturated state), TO (tonic), SWD, and the strength of stimulation (*A*_sps_) are indicated by text labels in each figure.

Secondly, in a similar way, we focus on the dynamical transitions when the system is in stable saturated states. We can see that single-pulse stimulation can not only suppress pathological firing patterns but also induce them. Low saturated state can transit to high saturated state, low-frequency clonic, low saturated state, and tonic oscillation after single-pulse stimulation acting on RE at *t* = 10 s, while high saturated state can only transit to itself, low-frequency clonic, and SWD (see Figure 8).

**Figure 9:**
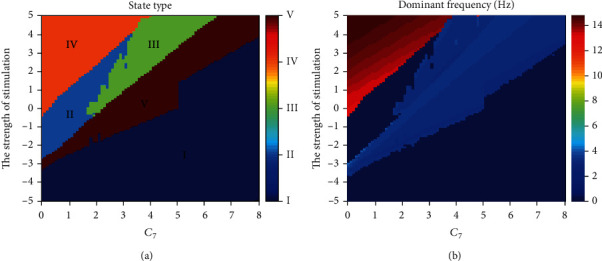
The state types (a) and dominant frequencies (b) after single-pulse stimulation acting on RE at *t* = 10 s when the system is in different initial states. Rich firing states such as I (high saturated state), II (low saturated state), III (SWD), IV (tonic), and V (low-frequency clonic) can be observed. The dominant frequencies of states I and II are 0 Hz and of states III and V are between 2 Hz and 4 Hz, while the dominant frequencies of state IV are nearly 14 Hz.

Finally, in order to systematically investigate how initial state and stimulus intensity affect state transitions, different firing states (see Figure 9(a)) and variations of corresponding dominant frequency (see Figure 9(b)) after single-pulse stimulation acting on RE are shown on the parameter plane (*C*_7_, the strength of stimulation). There are five different regions representing various effects of stimulation with the different initial states, which are denoted as I (high saturated state), II (low saturated state), III (SWD), IV (tonic), and V (low-frequency clonic). Specifically, if *C*_7_ ≤ 1.7, states I, V, II, and IV appear in succession as the strength of stimulation increases. For larger *C*_7_ (i.e., 1.7 < *C*_7_ ≤ 3.3), the system can display state III. In addition, states II and IV will disappear one after another when *C*_7_ is around 3.3 and 4, respectively. At last, when *C*_7_ ≥ 6.5, we can only observe two states, I and V.

### 3.3. Control of Deep Brain Stimulation

As one of the effective treatments for Parkinson's disease and epilepsy, deep brain stimulation (DBS) is more and more popular among scholars [57–59]. In this section, we focus on the effect of DBS control strategy on absence epilepsy. Similarly, stimulation is applied to RE, and we employ the typical period step function to simulate DBS as follows:

(3) $$\begin{array}{c} u\left(t\right)=A_{\text{DBS}}\times H\left(\sin\left(2\pi t/p\right)\right)\times \left(1-H\left(\sin\left(\frac{2\pi \left(t+\delta \right)}{p}\right)\right)\right), \end{array}$$

where *H* is the Heaviside bivalue step function and *A*_DBS_, *p*, and *δ* represent the amplitude, period, and pulse width of stimulus, respectively. Here, we set *p* = 0.1.

**Figure 10:**
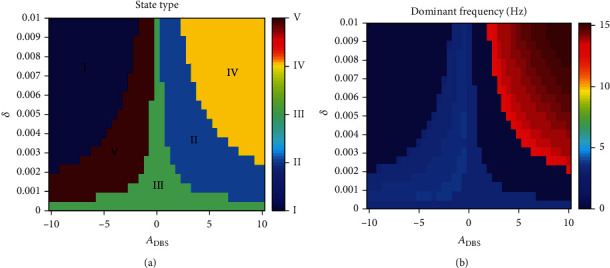
The state types (a) and dominant frequencies (b) after DBS acting on RE at *t* = 10 s when the system is in SWD originally. I (high saturated state), II (low saturated state), III (SWD), IV (tonic), and V (low-frequency clonic) can be observed. The dominant frequencies of states I and II are 0 Hz and of state III and V are between 2 Hz and 4 Hz, while the dominant frequencies of state IV are between 13 Hz and 15 Hz.

**Figure 11:**
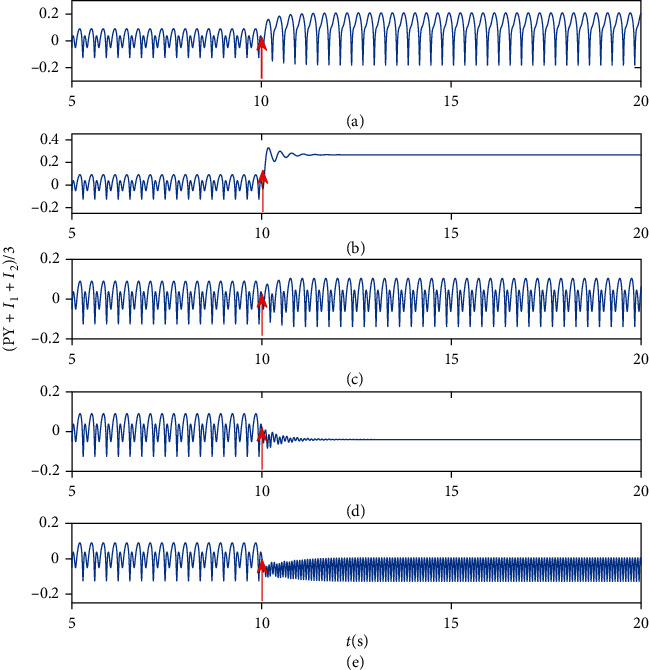
Transition behaviors under DBS acting on RE at *t* = 10 s, given by red arrows: (a) SWD to low-frequency clonic, A_DBS_ = −5, *δ* = 0.001; (b) SWD to high saturated state, *A*_DBS_ = −7, *δ* = 0.006; (c) SWD to SWD, *A*_DBS_ = 1, *δ* = 0.001; (d) SWD to low saturated state, A_DBS_ = 5, *δ* = 0.002; (e) SWD to tonic, *A*_DBS_ = 7, *δ* = 0.006. SWD can be controlled into normal or other pathological discharge behaviors by regulating the amplitude and pulse width of stimulation.

It is clear that absence epilepsy can be excellently controlled by DBS. On the parameter plane (*A*_DBS_, *δ*), five different firing patterns can be found, which are denoted by I (high saturated state), II (low saturated state), III (SWD), IV (tonic), and V (low-frequency clonic). To be specific, if we apply the positive DBS stimulus to RE at *t* = 10 s, then the initial state (i.e., SWD) can be firstly controlled to the low saturated state, and then, the system presents tonic oscillation as *A*_DBS_ and *δ* increase (see Figure 10). However, if DBS stimulus is negative, the system will transit from SWD to low-frequency clonic, then to high saturated state. Certainly, it is worthy to release absence seizure and stimulation side effect as few as possible and taking parameters from region II in Figure 10. In addition, we also specifically show the time series diagram of each area in Figure 10 (see Figure 11). We can clearly see five different transition behaviors, such as SWD to low-frequency clonic, SWD to high saturated state, SWD to SWD, SWD to low saturated state, and SWD to tonic (see Figure 11).

### 3.4. Control of Coordinated Reset Stimulation

Considering the side effect of single-pulse stimulation and DBS, here, we further explore the control effect of 3 : 2 CRS strategy on absence epilepsy, which is more effective and safer. The stimulus form is similar to the previous research [44], and the stimulation targets are TC and RE. In addition, in order to compare the effect of different stimulation strategies, we introduce two qualitative measurable indexes firstly. One is the percentage reduction (i.e., *η*) in absence seizures area, which can be described as below:

(4) $$\begin{array}{c} \eta =\frac{N_{1}-N_{2}}{N_{1}}, \end{array}$$

where*N*_1_ and *N*_2_ denote the mesh numbers occupied by SWD in the two-dimensional parametric plane (*C*_7_, *C*_11_) before and after applying CRS to stimulation targets.

And the other is current consumption, which can be expressed as follows:

(5) $$\begin{array}{c} I_{\text{current}}=\frac{1}{\sqrt{N}}∙{\left\|I_{\text{stim}}\left(t\right)\right\|}_{2}, \end{array}$$

where *I*_stim_(*t*), ‖∙‖_2_, and *N* represent the stimulus intensity on (RE, TC), two norms of *I*_stim_(*t*), and total time steps, respectively.

**Figure 12:**
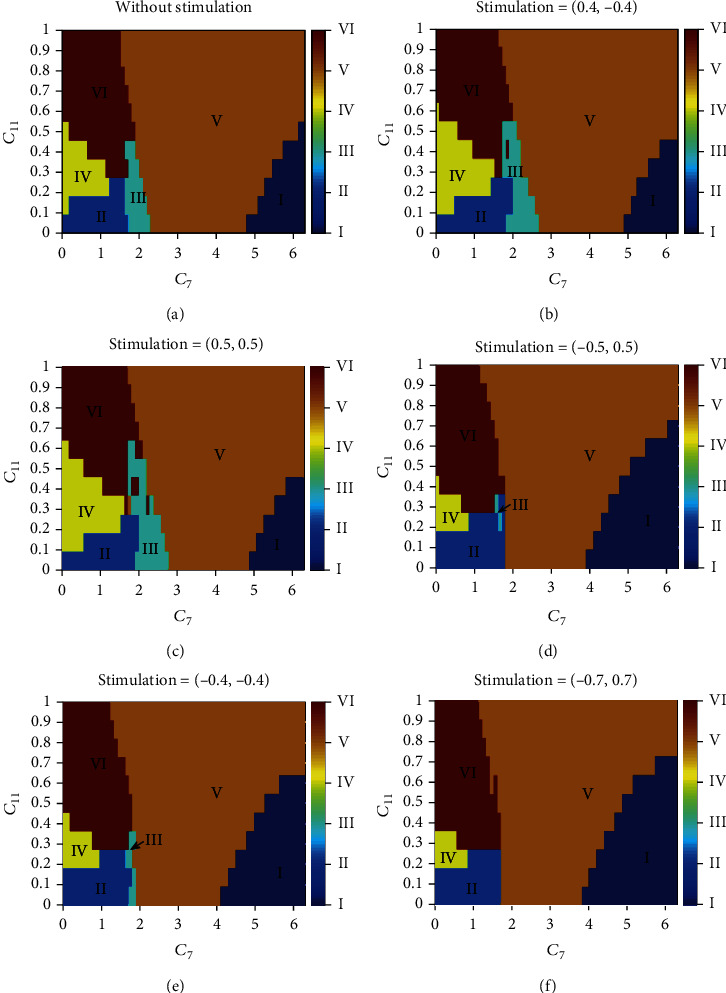
State types after applying CRS with different stimulus intensities on RE and TC, where I (high saturated state), II (low saturated state), III (SWD), IV (tonic), V (low-frequency clonic), and VI (high-frequency clonic) are six different firing states. (a–f) Is the two-dimensional parametric plane (*C*_7_, *C*_11_) without CRS. These typical two-dimensional plane diagrams show that SWD can be controlled well by CRS. And (-0.7, 0.7) is the most effective strategy for absence epilepsy.

**Figure 13:**
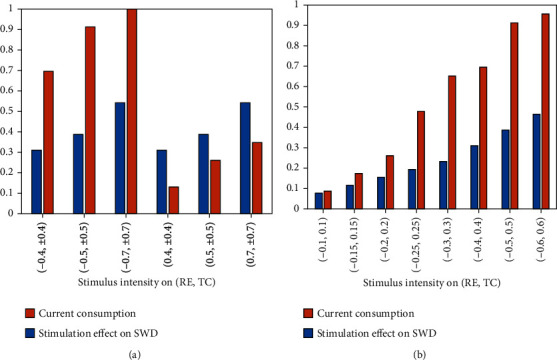
Stimulation effect on SWD and current consumption under different CRS stimulus intensities. The stimulus applied to RE can be positive or negative (a), while in (b), it is only negative. It can be seen from the bar graph that the greater the stimulation intensity, the best the control effect, although more and more energy is consumed.

Here, we set the frequency and pulse width of stimulus which are 100 Hz and 0.001 s. Since the accumulation of electric charge will cause side effects to patients, we firstly explore the strategies of applying the anodic pulse stimulus to RE and cathodic pulse stimulus to TC. For example, SWD region is reduced by about 13% when the stimulus intensity is (0.4, -0.4) (see Figure 12(b)). And SWD area will decrease as the stimulus amplitude increases (see Figure 13(a)). However, if we apply negative pulse stimulus to RE and positive pulse stimulus to TC, the control effect will be greatly improved, even up to 100% as the stimulus intensity is (-0.7, 0.7) (see Figure 12(f)). Then, we further verify that the greater the stimulus amplitude, the better the control effect (see Figure 13(b)). We also exhibit the control percentage *η* when the same nature of the stimulus is applied to our targets (see Figures 12(c) and 12(e)). Although SWD also can be controlled in this way, the accumulation of charge can cause some harm to the patient. Hence, we think that maybe (-0.7, 0.7) is a more effective strategy for absence epilepsy.

## 4. Discussions and Conclusions

It is demonstrated that there exists a basic disinhibition circuit among inhibitory neurons with different time scales [45]. And considering the fact that feedforward inhibition from TC has even more stronger functions on the cortex than the feedforward excitation in some cortical areas [46], we modify the classical Taylor neural field model to provide more theoretical foundation for the absence epilepsy treatment.

As described in many experimental results, astrocytes are involved in epilepsy [22–26]. The findings of the present study also highlight the potential of astrocyte as a useful complementary therapy for absence epilepsy. Rich transition dynamics can be induced by astrocyte dysfunction. If astrocytes are normal, the system will show normal low saturated state. However, when the function of astrocytes is inhibited or overexcited, pathological brain waves such as SWD, tonic, and clonic will appear.

Although single-pulse stimulation has improved in the last few years, the mechanisms are not entirely clear. At the same time, considering the important role of RE, we specifically show that when the system is in different initial states, the system can exhibit attractive dynamic transition with the increase of stimulus intensity after applying stimulation to RE. We find that single-pulse stimulation can not only suppress but also induce pathological firing patterns. Then, we applied DBS, a more clinically meaningful stimulation strategy to RE. The results suggest that DBS can control absence epilepsy by regulating the amplitude and pulse width of stimulation. Furthermore, we study another safer and more popular stimulation strategy, which is 3 : 2 CRS with different stimulation intensities. Our results show that the control effect on SWD will be greatly improved as the stimulus amplitude increases. However, results obtained through theoretical models still need to be compared with medical experiments and clinical practice in our future works.

It should be noted that we only studied the effect of a specific function of astrocytes on the pathological structure of neural networks, and the multiple-scale network models of the interneuron, pyramidal cell, and the astrocyte should be developed in our next work. In addition, more and more scholars pay attention to dynamic phenomena generated by random factors [60–62]. We hope to conduct a comprehensive study in this area.

## Figures and Tables

**Table 1:** Standard values of main model parameters.

Symbol	Value	Symbol	Value	Symbol	Value
*h*_PY_	-0.3	*ε*	2.5*e*+5	*C*_8_	2
*h*_*I*_1__	-3.4	*a*	2.8	*C*_9_	1
*h*_*I*_2__	-2	*b*	0.5	*C*_10_	2
*h*_TC_	-2.5	*C*_1_	1.8	*C*_11_	0.1
*h*_RE_	-4.5	*C*_2_	4	*C*_12_	0.05
*σ*_1_	26	*C*_3_	1.5	*C*_in1_	0.1
*σ*_2_	32.5	*C*_4_	0.1	*C*_in2_	0.3
*σ*_3_	30	*C*_5_	8	*C*_iny_	0.1
*σ*_4_	2.6	*C*_6_	1		
*σ*_5_	2.6	*C*_7_	2		

## Data Availability

The data used to support the findings of this study are included within the article.
